# 5,6,7,4’-Tetramethoxyflavanone protects against neuronal degeneration induced by dexamethasone by attenuating amyloidogenesis in mice

**DOI:** 10.17179/excli2019-1940

**Published:** 2020-01-02

**Authors:** Kanet Pakdeepak, Ratchanaporn Chokchaisiri, Jiraporn Tocharus, Pranglada Jearjaroen, Chainarong Tocharus, Apichart Suksamrarn

**Affiliations:** 1 Department of Physiology, Faculty of Medicine, Chiang Mai University, Chiang Mai 50200, Thailand; 2 Graduate School, Chiang Mai University, Chiang Mai 50200, Thailand; 3 Department of Chemistry, School of Science, University of Phayao, Phayao, Thailand; 4 Department of Anatomy, Faculty of Medicine, Chiang Mai University, Chiang Mai 50200, Thailand; 5 Department of Chemistry and Center of Excellence for Innovation in Chemistry, Faculty of Science, Ramkhamhaeng University, Bangkok 10240, Thailand

**Keywords:** 5,6,7,4'-tetramethoxyflavanon, Alzheimer's disease, amyloidogenesis, dexamethasone, neurodegeneration

## Abstract

Long-term exposure to high glucocorticoid levels induces memory impairment and neurodegeneration in Alzheimer's disease (AD) by increasing the expression of amyloid β and tau hyperphosphorylation (pTau). Previous studies showed beneficial effects of flavonoids in neurodegenerative models. 5,6,7,4'-tetramethoxyflavanone (TMF) is one of the active ingredients in *Chromolaena odorata* (L.), which R. M. King and H. Rob discovered in Thailand. This study focused on the effects of TMF on dexamethasone (DEX)-induced neurodegeneration, amyloidogenesis, pTau expression, neuron synaptic function, and cognitive impairment and the potential mechanisms involved. Mice were intraperitoneally administered DEX for 28 days before being treated with TMF for 30 days. The mice were randomly divided into six groups (twelve mice per group): control; TMF administration (40 mg/kg); pioglitazone administration (20 mg/kg); DEX administration (60 mg/kg); DEX administration plus TMF; and DEX administration plus pioglitazone. Behavioral tests showed that TMF significantly attenuated the memory impairment triggered by DEX. Consistently, TMF reduced DEX-induced amyloid beta production by reducing the expression of beta-site APP cleaving enzyme 1 (BACE1) and presenilin 1 (PS1), whereas it increased the gene expression of a disintegrin and metalloprotease 10 (ADAM10). TMF treatment also decreased pTau expression, inhibited phosphonuclear factor-kappa B (pNF-kB) and inhibited *glycogen synthase kinase 3* (GSK-3) activity by increasing GSK3 phosphorylation (pGSK3). In addition, TMF also improved synaptic function by increasing the expression of synaptophysin (Syn) and postsynaptic density protein 95 (PSD95) while decreasing acetylcholine esterase activity. Conclusively, TMF provided neuroprotection against DEX-induced neurodegeneration. These findings suggest that TMF might have potential as a therapeutic drug for AD.

## Introduction

Alzheimer's disease (AD) is a neurodegenerative disorder leading to memory loss (Burns and Iliffe, 2009[3]). AD pathology is related to an increase in amyloid beta (Aβ), which forms senile plaques, and hyperphosphorylated neuronal cytoskeleton protein (tau protein), which aggregates into a neurofibrillary tangle (NFT). Aβ is generated from the amyloid precursor protein (APP) by beta-site APP cleaving enzyme 1 (BACE1) and gamma secretase (O'Brien and Wong, 2011[29]). In addition, Aβ impairs neuronal synaptic function by decreasing the expression of both presynaptic molecules, such as synaptophysin (Syn), and postsynaptic proteins, such as postsynaptic density protein 95 (PSD95), to decrease neuronal function, decrease learning and memory behavior and induce loss of short-term memory (Liu et al., 2010[24]). Previous studies have demonstrated the involvement of the amyloidogenic pathway and high levels of glucocorticoids (GCs). Long-term exposure to high GC levels causes an AD-like pathology associated with an increase in Aβ production and hyperphosphorylated tau (pTau), which are the major causes of AD. Chronic stress increases GC secretion from the adrenal gland and binds with cytoplasmic glucocorticoid receptor (GR), resulting in GR activation; then, the active GR forms a homo-oligomer structure and translocates into the nucleus. In the nucleus, the GR oligomer activates APP and BACE-1 transcription by directly binding with the glucocorticoid response element (GRE) and regulates transcription to increase APP and β-secretase expression (Wang et al., 2011[47]). Moreover, the administration of dexamethasone (DEX) to a transgenic mouse model of AD increased NFT pathology (Joshi et al., 2013[17]). Tau protein stabilizes the axon microtubules of neurons and increases microtubule flexibility, leading to a healthy, functional neuron. Tau protein shows high expression in neurons but very low expression in astrocytes and oligodendrocytes (Billingsley and Kincaid, 1997[1]). Tau protein is phosphorylated by glycogen synthase kinase (GSK3) (Billingsley and Kincaid, 1997[1]; Taniguchi et al., 2001[43]) and is a key component of NFT. Therefore, activation of a disintegrin and metalloprotease 10 (ADAM10), inhibition of BACE1 and presenilin 1 (PS1) and reduction of NFT are potential treatments for AD. Pioglitazone is a peroxisome proliferator-activated receptor gamma (PPARγ) agonist. Many previous studies have demonstrated that pioglitazone has a pharmacological advantage in several AD models (Maeshiba et al., 1997[25]; Yan et al., 2003[50]). Pioglitazone has been shown to reduce Aβ plaques in APPV717I transgenic mice (Heneka et al., 2005[15]). In addition, pioglitazone also reduced the expression of BACE1 and Aβ1-42 and improved memory impairment in APP transgenic mice (Pedersen et al., 2006[33]; Sastre et al., 2003[36]; Sastre et al., 2006[37]). Thus, pioglitazone was used as a positive control in this study.

**Figure 1:**
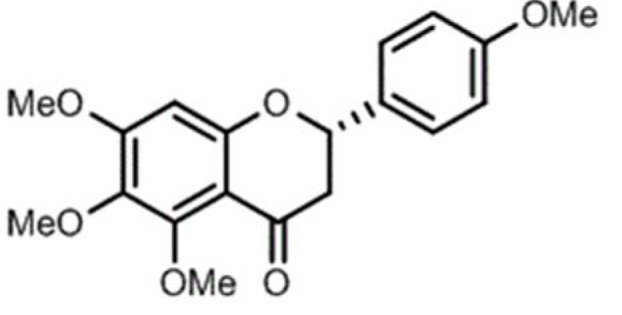
The chemical structure of 5,6,7,4'-tetramethoxyflavanone (TMF)

5,6,7,4'-tetramethoxyflavanone (TMF, Figure 1(Fig. 1)) is a flavonoid isolated from *Chromolaena odorata* (L.) by R.M. King & H. Rob. Flavonoids have a wide range of beneficial effects, including anti-oxidative and anti-inflammatory effects, and improves memory deficits in mice (Gonzalez-Gallego et al., 2010[13]; Panahi et al., 2016[32]; Kao et al., 2010[18]; Mori et al., 2012[26]). This study aimed to investigate the neuroprotective activity of TMF and explore the underlying mechanisms of action.

## Materials and Methods

### Chemicals and reagents

Dexamethasone sodium phosphate Lodexa-5® was purchased from L.B.S. Laboratory Ltd. (Bangkok, Thailand). Mouse anti-β-actin-HRP conjugate monoclonal antibody, rabbit antisera against phosphonuclear factor-kappa B (p-NF-κB) and rabbit polyclonal antisera against phospho-Tau (Ser202) were purchased from Cell Signaling Technology, Inc. (Danvers, MA, USA). Mouse monoclonal antibody against synaptophysin, mouse monoclonal antibody against PSD95, mouse monoclonal antibody against GSK3, mouse monoclonal antibody against phospho-GSK3 (Tyr279/Tyr216), rabbit antisera against active caspase 3 (cleaved form), goat polyclonal antisera against mouse IgG-peroxidase conjugate and mouse monoclonal antisera against rabbit IgG-peroxidase conjugate were purchased from EMD Millipore Corporation (Temecula, CA, USA).

### Isolation of TMF

Air-dried leaves of *C. odorata* (8.0 kg) were pulverized and extracted successively with n-hexane, EtOAc and MeOH at room temperature. The filtered solutions were evaporated to dryness under reduced pressure at 40-45 °C to obtain the hexane (663.24 g), EtOAc (956.7 g) and MeOH (546.25 g) extracts, respectively. The hexane extract (600.0 g), which has high neuroprotective activity in SK-N-SH cells, was fractionated by silica column chromatography. The first fraction contained fatty acids and nonpolar components. The second fraction (129.3 g) was chromatographed on a Sephadex LH-20 column, which was eluted with 40 % CH_2_Cl_2_ in MeOH to furnish 3 fractions, and the second fraction was crystallized from MeOH to give 5,6,7,4'-tetramethoxyflavanone (5.78 g) as the major constituent. The structure of this compound was confirmed by comparison of the spectroscopic data with the literature values (Suksamrarn et al., 2004[41]).

### Animals

Male ICR mice weighing 35-40 g (6-8 weeks old) were obtained from the National Laboratory Animal Center, Mahidol University, Salaya, Nakorn Pathom, Thailand. The mice were housed under a 12:12 h dark/light cycle at a constant temperature (25±1 °C) and ad libitum normal chow food and water. All experimental procedures were approved by the Institutional Animal Care and Use Committee at the Faculty of Medicine, Chiang Mai University, (Permit number: 30/2559) and performed in accordance with the National Institute of Health Guide for the Care and Use of Laboratory Animals.

### Animal treatment

The mice were randomly assigned to the following six groups (twelve mice per group): (1) control; (2) TMF administration (40 mg/kg); (3) pioglitazone administration (20 mg/kg); (4) DEX-administration (60 mg/kg); (5) DEX administration plus TMF; (6) DEX administration plus pioglitazone.

The mice in the DEX-administration group, the DEX-administration plus TMF group, and the DEX-administration plus pioglitazone group received daily intraperitoneal injections for 58 days. The mice in the DEX-administration plus TMF group, the pioglitazone group, the TMF (40 mg/kg) group and the pioglitazone (20 mg/kg) group received oral administration daily for 30 days starting from day 28 of the DEX injection. All control animals were given normal saline with the same volume via intraperitoneal injection and oral administration, respectively. After treatment, the mice were submitted to behavioral tests and then sacrificed by decapitation. The brain tissues were removed for further studies. RNA for qRT-PCR was extracted from the hippocampal region, and protein extraction was performed with the whole brain of mice in each group for the protein studies. 

### Open-field test

The open-field test is a method for measuring locomotion and anxiety (Seibenhener and Wooten, 2015[38]). The open-field apparatus size was 72 x 72 cm with a 36-cm wall. The mice were placed into the open-field apparatus at one corner and allowed to explore the apparatus for 5 min. After 5 min, the mice were returned to their home cages, and the open-field box was cleaned. The mice were exposed to the apparatus for two consecutive days. Every experiment was recorded by video camera and analyzed using the SMART system (Panlab Harward Apparatus; Bioscience Company, MA, USA).

### Morris water maze (MWM) test

Spatial memory was assessed using the Morris water maze (MWM) test (Vorhees and Williams, 2006[46]), which consisted of a five-day location navigation training and a probe test on day six. The maze was a circular pool (100 cm in radius and 40 cm high) that was filled with water at a temperature of approximately 23±1 °C. The tank was divided into four quadrants, one of which contained a circular platform (10 cm diameter) placed at a fixed position two cm below the surface of the water. Oriented navigation trials were performed four times per day for five consecutive days with a constant interval of one hour. For all training trials, the time that it took the mouse to reach the submerged platform (escape latency) was recorded to assess spatial learning ability. On the sixth day, another set of tests consisting of a trial with the hidden platform was conducted. The time required for individual mice to find the platform was two minutes. The time required to swim to the target quadrant and the swimming distance were recorded using SMART video tracking software (Panlab Harward Apparatus; Bioscience Company, Holliston, MA, USA).

### Measurement of secretase enzyme by real-time quantitative polymerase chain reaction (qRT-PCR) 

Total RNA was isolated from the hippocampal region for each group using TRIzol reagent (MACHEREY-NAGEL GmbH and Co. KG, Düren, Germany). For all RNA samples, the purity and quantity were determined with a Nanodrop. One microgram of total RNA was used for cDNA synthesis with a ReverTraAce® qPCR RT kit (Toyobo, Tokyo, Japan). PCR amplification was performed using a KOD SYBR qPCR kit (Toyobo, Tokyo, Japan) with an ABI PRISM 7500 Sequence Detection system (Applied Biosystems, Foster City, CA, USA). Primer sets specific for PS1, BACE1, ADAM10, APP and GAPDH were designed. The primers sequences are shown below. 

**PS1:**

Forward: 5'GACGGTCAGCTAATCTACAC3'

Reverse: 5'GATAAATACCAGGGCCATGAG3'

**BACE1:**

Forward: 5'TGTGCCCTACACCCAG3'

Reverse: 5'GTATAGCGAGTGGTCGAT3'

**ADAM10:**

Forward: 5'GCCAGCCTATCTGTGGAAACGGG3'

Reverse: 5'TTAGCGTCGCATGTGTCCCATTTG3'

**APP:**

Forward: 5'GCTGCCCAGCTTGGCACTGC3'

Reverse: 5'GGCAACGGTAAGGAATCACGATGTGGGTG3'

**GAPDH:**

Forward: 5'AGAAGGTGGTGAAGCAGGCATC3'

Reverse: 5'CGAAGGTGGAAGAGTGGGAGTTG3'

### Histological analysis by hematoxylin and eosin (H&E) staining

The coronal sections were prepared and stained with hematoxylin and eosin (H&E) to observe the morphological changes. After treatment, the mouse brain tissues were immediately removed and fixed in 10 % formalin in phosphate-buffered saline (PBS), pH 7.4, and embedded in paraffin. Then, the coronal brain tissues were sectioned at 20-μm thickness using a microtome (Leica, Germany) and stained with H&E for observation pathological changes under a light microscope (Olympus AX70, Tokyo, Japan).

### Terminal deoxynucleotidyl transferase UTP nick end labeling (TUNEL) assay

TUNEL staining was used to detect cell apoptosis based on DNA fragmentation caused by apoptotic signaling cascades. The brain tissues were fixed in 4 % formaldehyde, embedded in paraffin, and sectioned at a thickness of 4 µm. The sections were deparaffinized and rehydrated. Then, TUNEL staining was performed according to the manufacturer's instructions with a TUNEL assay kit (Roche Diagnostics GmbH, Mannheim, Germany). Finally, the total number of cells and TUNEL-positive cells were observed under light microscopy. Five high-power fields (×400) were randomly selected, and the number of apoptotic cells was counted in each field. The apoptosis index (AI) = number of positive cells per number of total cells.

### Acetylcholinesterase (AChE) level

Brain tissues were collected and placed in lysis buffer. Brain homogenates were incubated with 100 µl of Ellman's reagent and 190 µl of 75 mM propionyl thiocholine iodide at 37°C for 20 min. Absorbance values were read at 405 nm in a microplate reader every 5 sec for 2 min.

### Western blotting

After treatment, brain tissues were collected and homogenized with lysis buffer (1.5 mM MgCl_2_, 10 mM KCl, 20 mM HEPES, 1 mM EDTA, 1 mM EGTA, 250 mM sucrose, 0.1 mM phenylmethylsulfonyl fluoride, 1 mM dithiothreitol, and proteinase inhibitor; pH 7.9). The homogenate was centrifuged at 10,000 rpm for 15 min at 4 °C, and the supernatant was collected. The protein concentration was measured using a Bradford assay with bovine serum albumin (BSA) as the standard. Equal amounts of protein sample were loaded in each well of a 10-15 % sodium dodecylsulfate-polyacrylamide gel for SDS-PAGE. The proteins were transferred to a polyvinylidine fluoride (PVDF) membrane (Immobilon-P, Millipore, Bedford, MA, USA), which was incubated with 5 % nonfat milk at 4 °C overnight.

The membrane was then incubated with the following primary antibodies: rabbit anti-pTau (1:1,000, Cell Signaling); mouse anti-GSK3 (1:1,000, Millipore); mouse anti-pGSK3 (1:1,000, Millipore); mouse anti-PSD95 (1:1,000, Millipore); mouse anti-synaptophysin (1:1,000, Millipore); rabbit anti-cleaved caspase 3 (1:1,000, Millipore); and anti-phospho NF-kB and mouse anti-beta-actin (1:1,000, Cell Signaling) at 4 °C overnight. The membrane was washed three times with TBST and then incubated with horseradish peroxidase-conjugated secondary antibody for 2 h at room temperature. The chemiluminescent bands were visualized using ECL substrate solution (Millipore, MA, USA) before being exposed to X-ray films. The densitometric analysis was performed using a scanning densitometer and analyzed using ImageJ software (National Institute of Health, Bethesda, MD, USA).

### Statistical analysis

Animal behavioral data were analyzed using two-way analysis of variance (ANOVA) with repeated measures followed by a post hoc test. All other values are presented as the mean ± SEM. The significance of the difference between the means of the two groups was analyzed by one-way analysis of variance (ANOVA) and Tukey's post‐hoc multiple test for comparisons among the experimental groups. A value of *p *< 0.05 was considered significant.

## Results

### Effects of TMF on anxiety behavior after chronic DEX exposure

**Figure 2:**
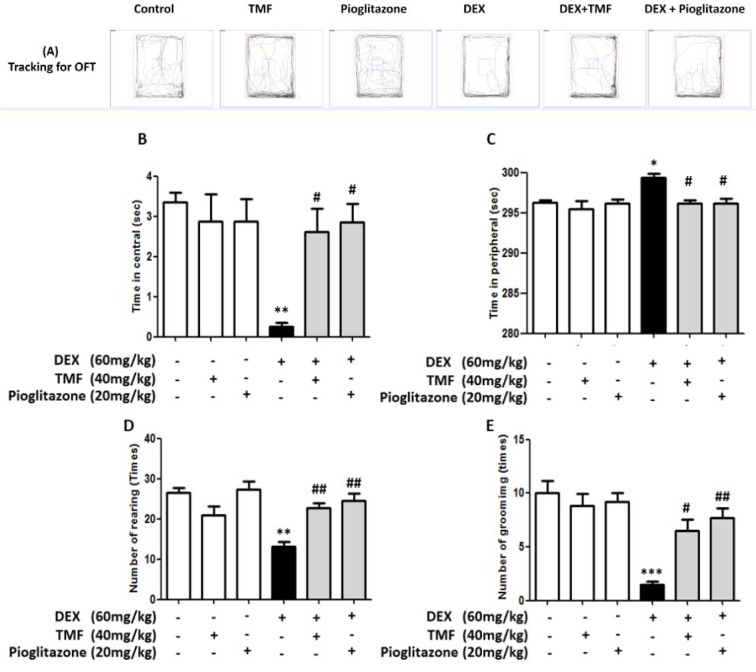
The effect of TMF on anxiety behavioral outcomes was investigated by open-field test (OFT). (A) The tracking for the OFT by the SMART program. (B) The time spent in central area of the open-field box. (C) The time spent in periphery of the open-field box. (D) The frequency of rearing behavior in 5 min. (E) The frequency of grooming behavior in 5 min. The data are the mean ± SEM from three independent experiments (**p<0.01, ***p<0.001 compared with the control group, ^#^p<0.05, ^##^p<0.01 compared with the DEX group).

The anxiety behavior in mice chronically exposed to DEX and treated with TMF at a dosage of 40 mg/kg for 30 days was evaluated using the open-field test (Figure 2A(Fig. 2)). DEX treatment significantly decreased the time spent in the central area compared with that in the control group (*p*< 0.01), but there was no significant difference in the TMF and pioglitazone treatment groups compared with the control group. TMF treatment in DEX mice resulted in a significantly increased time in the central area compared with that of DEX group at a level similar to that of the pioglitazone group (*p*< 0.05) (Figure 2B(Fig. 2)). The time spent in the periphery was analyzed and is shown in Figure 2C(Fig. 2). The DEX group showed significantly increased time in the periphery compared with the control group (*p*<0.05). No differences were evident in the TMF and pioglitazone groups compared with the control group. The DEX plus TMF or DEX plus pioglitazone groups showed a significant decrease in the time spent in the periphery compared with the DEX group (*p*<0.05). Anxiety behavioral outcomes were investigated by observing the frequency of rearing and grooming, as shown in Figure 2D(Fig. 2) and Figure 2E(Fig. 2). The frequency of both behaviors was significantly decreased in the DEX group compared with that in the control group (*p*<0.01 and* p*<0.001, respectively), but no differences were observed in the TMF and pioglitazone groups compared with the control group. Significantly increased rearing and grooming were found in the DEX plus TMF group (*p*<0.01 and *p*<0.05). The DEX plus pioglitazone group was also found to exhibit significant increases in both behaviors (*p*<0.01).

### TMF treatment suppresses the cognitive impairment induced by DEX in mice 

**Figure 3:**
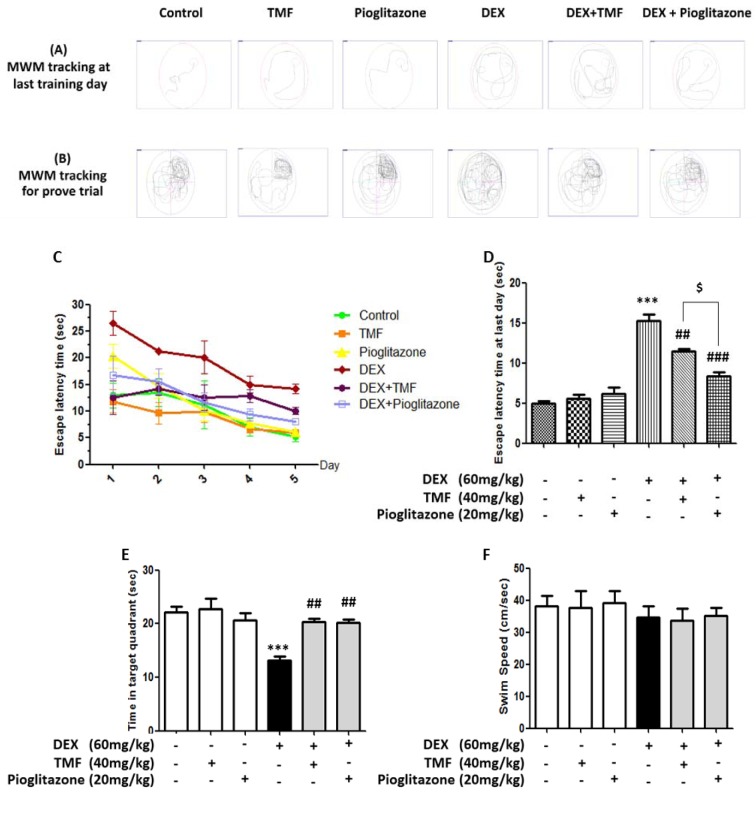
The effect of TMF on learning and memory behavior performance was investigated by Morris water maze test. (A) The tracking for the last training day by the SMART program. (B) The tracking for the trial test. (C) Escape latency time for 5 days. (D) Escape latency time on the last day. (E) Time spent in target quadrant during the probe trial. (F) Swimming speed. The data are the mean ± SEM from three independent experiments (***p<0.001 compared with the control group, ^##^*p*<0.01, ^###^*p*<0.001 compared with the DEX group, ^$^*p*<0.05 compared with pioglitazone treatment in the DEX administration group).

The spatial learning and memory ability of the mice were evaluated by the Morris Water Maze Test. The tracking for MWM on the last training day is shown in Figure 3A(Fig. 3), and the tracking for the trial test is shown in Figure 3B(Fig. 3). Figure 3C-D(Fig. 3) show that the mice in the DEX-administration group had a significant impairment in spatial learning ability during the 5-day test, as demonstrated by the longer time needed to find the platform compared with the control group. A significant difference in escape latency time was observed in the DEX plus TMF treatment group and the DEX plus pioglitazone group on the last training day compared with that in the DEX group (*p*<0.01, *p*<0.001, respectively). Pioglitazone treatment with DEX administration also resulted in a significant decrease in the time needed to find the platform compared with TMF treatment with DEX administration (*p*<0.05). The hidden platform was removed to perform a probe trial. As shown in Figure 3E(Fig. 3), the DEX-administration group showed a significant decrease in the time spent in the target quadrant (*p*<0.001). However, the mice in the DEX administration plus TMF group or the pioglitazone group displayed remarkable increases in the time spent in the target quadrant compared with those in the DEX group (*p<*0.01, *p*<0.01, respectively). In addition, in normal mice, the administration of TMF or pioglitazone did not show any effects compared with the control group. The swimming speed in each group is shown in Figure 3F(Fig. 3); the results showed no significant differences between the groups. The results indicate that DEX-induced mice have an impairment in spatial learning and memory, while TMF or pioglitazone treatment could restore cognitive function.

### Effects of TMF on amyloidogenesis in mouse brain

**Figure 4:**
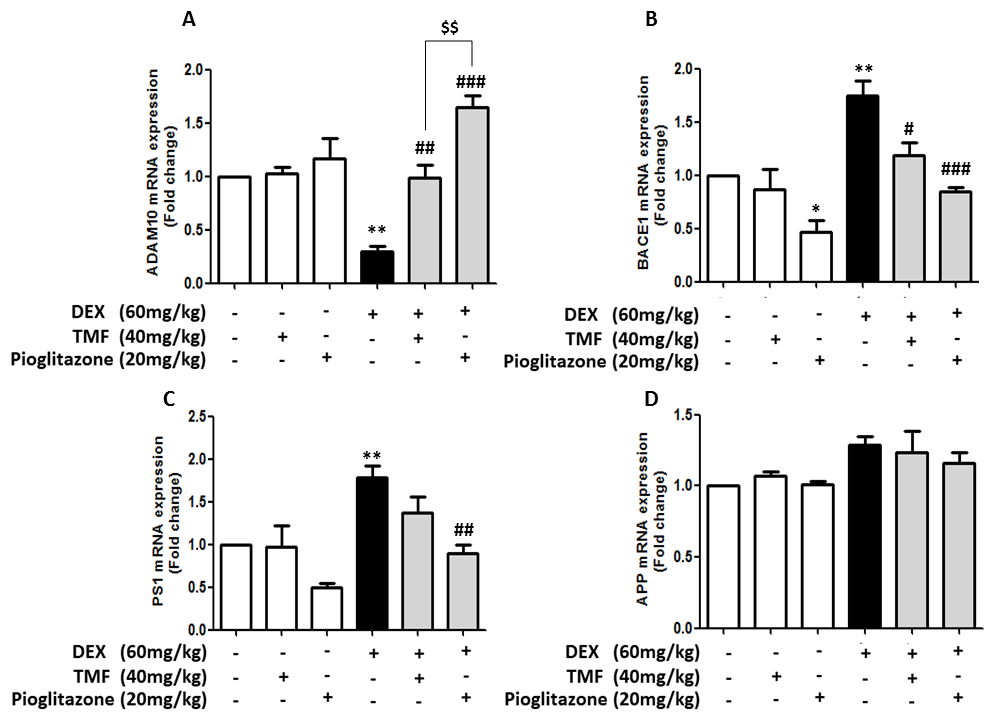
RT-PCR performance to investigate the effect of TMF on amyloidogenic-related gene expression. The data were analyzed as the fold change of mRNA expression of ADAM10 expression (A), BACE1 (B), PS1 (C) and APP (D). The data are the mean ± SEM from three independent experiments (*p<0.05, **p<0.01 compared with the control group, ^#^*p*<0.05, ^##^*p*<0.01, ^###^*p*<0.001 compared with the DEX group, ^$$^*p*<0.01 compared with pioglitazone treatment in the DEX administration group).

Amyloidogenesis involves gene expression related to amyloid beta (Aβ) production, leading to memory impairment pathology. The effects of TMF on amyloidogenic-related gene expression were investigated for the genes ADAM10, BACE1, PS1 and APP by real time-polymerase chain reaction (RT-PCR) (Figure 4(Fig. 4)). The expression of the ADAM10 gene was significantly decreased in the DEX group compared with that in the control group (*p*<0.01). There was no significant difference between the TMF or pioglitazone groups and the control group. The mice in the DEX administration plus TMF group or the pioglitazone group displayed remarkably increased ADAM10 gene expression compared with the DEX group (*p*<0.01 and *p*<0.001). Interestingly, the DEX administration plus pioglitazone group showed a significant increase in the ADAM10 gene compared with the DEX administration plus TMF group (*p*<0.001). The DEX group showed a significant increase in BACE1 and PS1 gene expression (*p*<0.01) compared with the control group. The mice in the DEX administration plus TMF group had significantly decreased BACE1 gene expression (*p*<0.05) but did not show a significant difference in PS1 gene expression compared with the DEX group. Interestingly, the mice in the DEX administration plus pioglitazone group had significantly decreased BACE1 and PS1 gene expression (*p*<0.001 and *p*<0.01) compared with the DEX group (Figure 4B, C(Fig. 4)). However, APP gene expression was not found to be significantly different between the groups (Figure 4D(Fig. 4)).

### TMF attenuates Tau hyperphosphorylation (pTau) expression in DEX-induced mice

**Figure 5:**
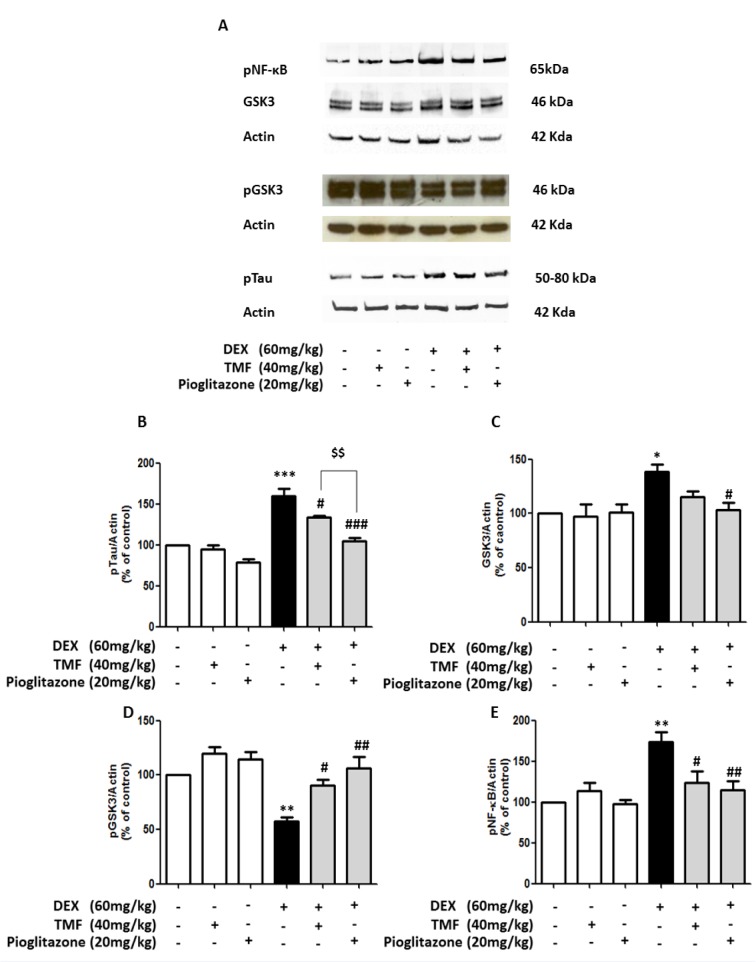
The attenuation effect of TMF on pTau expression. (A) Representative of GSK3, pGSK3, pNF-κB and pTau protein expression as determined by Western blotting. Brain tissues were used for electrophoresis and immune blotting. The bands were analyzed and are presented as a histogram, with actin as the control. (B) The quantitative results of all groups for pTau expression. (C) The quantitative results of all groups for GSK3 expression. (D) The quantitative results of all groups for pGSK3 expression. (E) The quantitative results of all groups for pNF-κB expression. The data are the mean ± SEM from three independent experiments (*p<0.01, **p<0.01, ***p<0.001 compared with the control group, ^#^*p*<0.05,^ ##^*p*<0.01, ^###^*p*<0.001 compared with the DEX group, ^$$^*p*<0.01 compared with pioglitazone treatment in the DEX administration group).

pTau levels were investigated by Western blotting (Figure 5A-B(Fig. 5)). pTau levels were significantly increased in the DEX group compared with the control group (*p*<0.001) and did not show significant differences in the TMF or pioglitazone treated normal mice compared with the control group. Treatment with TMF and pioglitazone in the DEX administration group had significantly decreased pTau levels compared with the DEX group (*p*<0.05 and *p<*0.001). However, there was a significant decrease in pTau in the pioglitazone treatment in DEX administration group compared with the TMF treatment in DEX administration group (*p<*0.01). 

We next further investigated the effect of TMF on GSK3, pGSK3 and nuclear factor-kappa B phosphorylation (pNF-kB) expression by Western blot, as shown in Figure 5C, D and E(Fig. 5), respectively. The DEX group significantly increased GSK3 expression (*p*<0.05) and decreased the pGSK3 level (*p*<0.01) compared with the control group. The TMF or pioglitazone treated normal mice did not show a significant difference in GSK3 expression or pGSK3 levels compared with the control group. The DEX administration mice plus TMF had a significantly increased pGSK3 level (*p*<0.05); however, there was no significant difference in GSK3 expression compared with the DEX group. Moreover, GSK3 expression was significantly decreased in mice administered DEX plus pioglitazone compared with the DEX group (*p*<0.05). pGSK3 levels were significantly increased in the DEX administration mice plus pioglitazone (*p*<0.01) compared with the DEX group. Moreover, pNF-kB was also significantly reduced in the DEX administration plus TMF or pioglitazone groups (*p<*0.05 and *p*<0.01, respectively).

### Effect of TMF on neuronal morphology change and neuronal apoptosis

**Figure 6:**
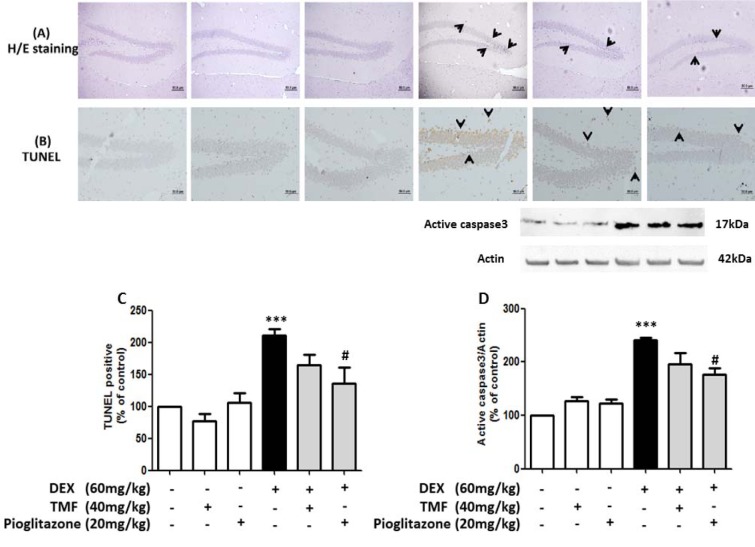
TMF attenuates neuronal apoptosis. Brain tissues were stained with H&E to determine neuronal morphology in the hippocampus region (A). TUNEL assay was used to investigate cell apoptosis in brain tissues (B), TUNEL-positive cells were analyzed as % of control to compare all groups (C). Representative cleaved caspase 3 levels as determined by Western blotting are presented in a histogram (with actin as the control) (D). The data are the mean ± SEM from three independent experiments (***p<0.001 compared with the control group, ^#^*p*<0.05 compared with the DEX group).

Neuronal morphology changes were evaluated by H&E staining (Figure 6A(Fig. 6)). The hippocampus area from the DEX group showed markedly increased pyknotic nuclei compared with the control group (black arrow). Conversely, a reduction of neuronal positive pyknotic nuclei was observed in the mice in the DEX plus TMF group or the pioglitazone group compared with the DEX group. Next, we investigated neuronal cell apoptosis utilizing a TUNEL assay to stain apoptotic neurons dark brown (Figure 6B, C(Fig. 6)). The neuronal cells in the hippocampus showed an increase in TUNEL-positive cells (black arrow) in the DEX group compared with the control (p<0.001). A decrease in TUNEL-positive cells was found in mice administered DEX plus TMF or pioglitazone. To determine neuronal cell apoptosis, the TUNEL-positive cells were counted and analyzed as percentages of the control as shown in Figure 6C(Fig. 6). The DEX group showed a significant increase in TUNEL-positive cells compared with the control group (*p*<0.001) but there was no significant difference in the TMF and pioglitazone group compared with the control group. A significant decrease in TUNEL-positive cells was found in the DEX plus pioglitazone group (*p*<0.05) compared with the DEX group. DEX plus TMF induced a decrease in TUNEL-positive cells compared with the DEX group; however, the difference was not significant. 

The expression of cleaved caspase3, which is known to be a crucial mediator of apoptosis, was determined by Western blotting (Figure 6D(Fig. 6)). DEX led to an increase in cleaved caspase 3 levels compared with the control group (*p*<0.001). TMF or pioglitazone treatment in normal mice did not induce significant differences in cleaved caspase 3 levels compared with the control group. TMF treatment in mice administered DEX reduced cleaved caspase 3 levels but showed no significant differences compared with the DEX group. A significant reduction of cleaved caspase 3 levels was found in mice administered with DEX plus pioglitazone compared with DEX (*p*<0.05).

### TMF improved neuronal synaptic function in DEX-induced mice 

**Figure 7:**
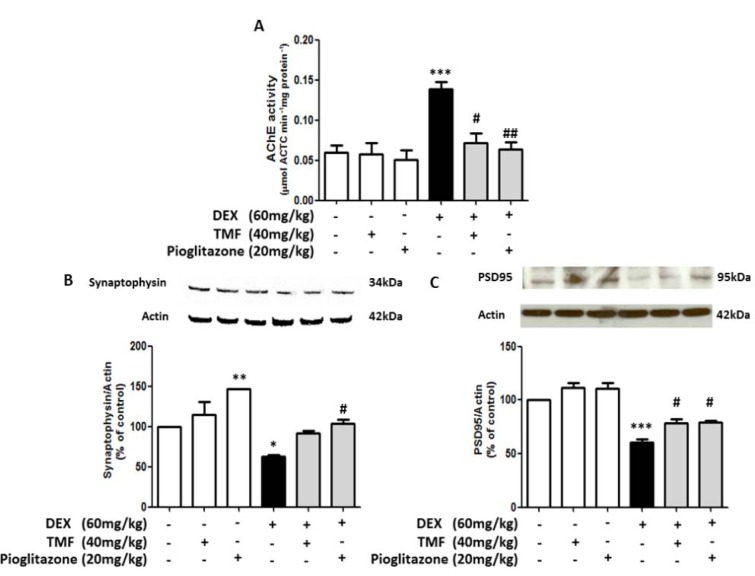
Improvement effect of TMF on neuronal synaptic function in memory-impaired mice. (A) Acetylcholinesterase activity assay in all groups. (B) and (C) Synaptophysin and PSD95 protein expression as determined by Western blotting; the results are presented as a quantitative histogram. The data are the mean ± SEM from three independent experiments (*p<0.01, ***p<0.001 compared with the control group, ^#^*p*<0.05, ^##^*p*<0.01, ^###^* p*<0.001 compared with the DEX group).

AChE activity was investigated in the brain in all groups (Figure 7A(Fig. 7)). AChE activity in the TMF or pioglitazone group was not significantly different; however, AChE activity was significantly increased in the DEX group compared with the control group (*p*<0.001). Treatment with TMF in DEX-induced mice induced a significant decrease AChE activity (p<0.05) compared with the DEX group. In addition, treatment with pioglitazone in DEX-induced mice induced a significant decrease in AChE activity (*p*<0.01). Expression of the presynaptic protein synaptophysin and the postsynaptic density protein PSD95 was determined by Western blot (Figure 7B-C(Fig. 7)). The results showed that both proteins were decreased in the DEX group compared with the control group. Interestingly, pioglitazone treatment in normal mice also induced a significant increase in synaptophysin expression (*p*<0.01) compared with the control. TMF treatment in DEX-induced mice significantly increased the levels of the post-synaptic protein PSD95 (p<0.05), while the levels of the presynaptic protein synaptophysin tended to increase, but not significantly, compared with the DEX group. Pioglitazone treatment in DEX-induced mice also significantly increased the levels of both the presynaptic protein synaptophysin (*p*<0.05) and the postsynaptic protein PSD95 (*p*<0.05) compared with those in the DEX group.

For more results see the Supplementary data.

## Discussion

Long-term exposure to high GC levels causes AD-like pathology by increasing neurodegeneration, which is a term that describes the progressive loss of the structure or function of neurons (Bossy-Wetzel et al., 2004[2]). The evidence for the relationship between chronic GC exposure and AD showed that the increase in Aβ and pTau expression caused memory deficits (Green et al., 2006[14]). Moreover, chronic GC exposure leads to neurodegeneration in the brain cortex and hippocampus (Hu et al., 2016[16]). DEX is a steroid drug widely used in inflammatory diseases. The structure of DEX is similar to that of GC; their action is through binding GR, and they also show effects similar to those of GC, including neurodegeneration. Several studies have demonstrated that DEX induces an AD model in rodents by increasing pTau, Aβ expression, and memory impairment (Li et al., 2012[23]; Tongjaroenbuangam et al., 2013[45]; Lesuis et al., 2018[22]; Ouanes and Popp, 2019[31]). This study aimed to study the neuroprotective effects of TMF and its possible mechanisms in DEX-induced mice. TMF alleviated DEX-stimulated amyloidogenesis and neuronal cell death as well as maintaining the balance of the cholinergic system, and such effects might be related to cognitive function enhancement.

The behavioral study demonstrated that learning and memory impairment was observed in mice exposed to DEX, whereas TMF treatment attenuated the deterioration of spatial learning and memory triggered by DEX. GC induces neurodegeneration associated with an increase in Aβ production, which is a major cause of AD (Murphy and LeVine, 2010[27]). The aggregates of Aβ peptide are a pathological hallmark of AD. The production of the Aβ peptide is through the activity of the BACE1 and PS1, which are widely considered to have a crucial role in initiating AD pathology (O'Brien and Wong, 2011[29]). Overexpression of BACE1 in the brain regions was observed in AD patients. Several lines of evidence have demonstrated that a reduction of BACE1 resulted in decreased Aβ production in several mouse models of AD (Kim et al., 2018[19]; Cai et al., 2012[4]; Xue et al, 2015[48]). Aβ acts as a major trigger of neuronal and glial pro-inflammatory activation. Following activation, both neuronal cells and glial cells produced inflammatory mediators, such as *tumor necrosis factor-α* (TNF-α), interleukin 1, beta (IL-1β), nitric oxide (NO) and cyclooxygenases (COXs), which was mediated by NF-κB (Ferrera et al., 2014[10]; Carrero et al., 2012[5]). Increased NF-κB activity plays a central role in regulating the expression of inflammatory genes. Upon activation, the IκBα protein, which is the inhibitory subunit of the NF-κB, undergoes phosphorylation and degradation, which enables the translocation of the NF-κB p65 subunit from the cytosol into the nucleus. Several lines of evidence have demonstrated that inflammation has an important role in the amyloidogenesis pathway via activating NF-κB signaling. Inflammation has been reported to result in increased APP processing and the accumulation of Aβ peptides (Ringheim et al., 1998[34]; Yamamoto et al., 2007[49]). NF-κB activates the BACE1 promoter and upregulates the expression of BACE1 (Chen et al., 2012[8]). In addition, NF-κB also regulates the transcriptional activity of APP and gamma-secretase promoters (Chami et al., 2012[6]). Previous studies have reported that chronic GC exposure induced neuroinflammation by activating NLR family pyrin domain containing 1 (NLRP1) inflammasome in brain tissues and inducing neuronal cell death (Zhang et al., 2017[51]). Several studies have reported that GC activates transcription to increase APP and β-secretase expression (Sotiropoulos et al., 2008[40]; Green et al., 2006[14]). Previous studies have demonstrated that the anti-inflammatory activity of flavonoids from plants occurs through suppressing NF-κB signaling (Naik et al., 2017[28]; Ruiz et al., 2007[35]). Therefore, suppressing NF-κB signaling in AD could prevent the amyloidogenesis pathway and neurodegeneration. We present evidence that DEX markedly upregulated BACE1 and PS1 and downregulated ADAM10 mRNA levels and reduced the accumulation of Aβ in brain tissues. TMF significantly increased the expression of α-secretase and decreased the production of the β-secretase without altering APP levels. Our results demonstrated that TMF decreased pNF-κB and the production of NO. Therefore, there is a possibility that the TMF stimulated the non-amyloidogenic processing of APP, and enhanced α-secretase levels also might decrease BACE1 expression through the negative regulation of NF-κB. 

GSK3 is an important kinase enzyme leading to the regulation of Aβ accumulation and pTau (Terwel et al., 2008[44]; DaRocha-Souto et al., 2012[9]). Abnormal tau protein induces the loss of their functions, disassociating from microtubules, leading to destabilization of the microtubules, the disruption of nerve impulses and loss of memory formation (Chatterjee et al., 2009[7]; Lambourne et al., 2005[20]). Moreover, pTau bound together generates NFT and aggregated in neurons, activating the apoptosis pathway and resulting in neuronal cell death (Lee et al., 2005[21]). Inhibition of GSK3 reduced tau phosphorylation. DEX upregulated pTau expression by activating GSK3 and down regulating pGSK3 expression. TMF tends to decrease GSK3 expression but significantly increased pGSK3 expression and significantly decreased pTau. Our results also demonstrated that TMF attenuated pTau and pGSK3 levels in mice treated with DEX, which indicated that TMF reduced tau phosphorylation, which may be associated with the GSK-3 signaling pathway.

Recent studies have demonstrated that an imbalance of the cholinergic system in the central nervous system (CNS), which is related to learning and memory impairment, occurred during amyloidogenesis (Garcia-Alloza et al., 2005[11]; Liu et al., 2010[24]). Neuron synaptic function was reduced in AD patients, which was related to a decrease in neurotransmitters, such as ACh, by increasing AChE activities (Garcia-Ayllon et al., 2010[12]). Moreover, AD patients also show changes in synaptic molecular expression that are important to neuronal synaptic function, especially, presynaptic molecules, such as synaptophysin, and postsynaptic proteins, such as PSD95 (Shao et al., 2011[39]; Tampellini et al., 2010[42]). This study found that DEX caused a decrease in ACh levels via elevating AChE activity. However, TMF treatment markedly normalized the cholinergic system in the mouse brain. We next determined the expression of both synaptic molecules. DEX decreased both synaptophysin and PSD95 expression levels, while TMF treatment upregulated the expression of PSD95. However, TMF tended to increase synaptophysin expression with no significant difference compared with DEX treatment alone. 

Many studies have suggested that apoptosis plays an important role in AD. Apoptosis leads to progressive neuronal cell loss in AD pathogenesis (Obulesu et al., 2014[30]). DEX treatment induced neuronal cell damage in several brain areas, including the cortex and hippocampus. Interestingly, TMF rescued the loss and damage of neurons triggered by DEX and enhanced cognitive ability in mice. Brain histology demonstrated that the neuronal cell morphology was changed in DEX mice, and TMF treatment induced improvement. Our results also showed markedly increased TUNEL-positive cells in DEX mice and a small number of TUNEL-positive cells in the brains of the TMF-treated group. Furthermore, TMF also decreased the levels of cleaved caspase-3 protein in DEX-mice. These results are related to the decrease in the number of apoptotic cells, clearly indicating the anti-apoptotic effects of TMF in DEX mice.

## Conclusions

In conclusion, it is evident that TMF treatment improves learning and memory impairment in DEX-induced mice by decreasing Aβ production and reducing pTau expression, resulting in increased neuronal cell survival. Moreover, TMF improved neuronal cell synaptic function by reducing AChE activity and increasing the expression of pre- and postsynaptic molecules. In addition, our study is the first to report the effect of TMF in DEX-induced mice. TMF might play a role in improving learning and behavior and may offer neuroprotection against AD.

## Compliance with Ethical Standards

### Conflict of interest

The authors declare that there is no conflict of interest.

### Acknowledgments

This study was supported by the Chiang Mai University, Functional Food Research Center for Well-Being, Chiang Mai University, and the Faculty of Medicine, Chiang Mai University. Support from The Thailand Research Fund (DBG6180030) and the Center of Excellence for Innovation in Chemistry, Ministry of Higher Education, Science, Research, and Innovation is gratefully acknowledged. Kanet Pakdeepak acknowledges financial support from the Graduate School, Faculty of Medicine, Chiang Mai University, and a graduate scholarship for 2019 from the National Research Council of Thailand (NRCT).

## Supplementary Material

Supplementary data
