# Supplementary material for: 5,6,7,4’-Tetramethoxyflavanone protects against neuronal degeneration induced by dexamethasone by attenuating amyloidogenesis in mice
Source: EXCLI J. 2020 Jan 2;19:16–32. doi: 10.17179/excli2019-1940 (PMC7003641; doi:10.17179/excli2019-1940)
Supplement: Supplementary data [file EXCLI-19-16-s-001.pdf]

EXCLI Journal 2020;19:16-32 – ISSN 1611-2156
Received: October 31, 2019, accepted: December 04, 2019, published: January 02, 2020

Supplementary data to:

5,6,7,4'-TETRAMETHOXYFLAVANONE PROTECTS AGAINST NEURONAL DEGENERATION INDUCED BY DEXAMETHASONE BY ATTENUATING AMYLOIDOGENESIS IN MICE

Kanet Pakdeepak[1,2], Ratchanaporn Chokchaisiri[3], Jiraporn Tocharus[1,*], Pranglada Jearjaroen[2], Chainarong Tocharus[4], Apichart Suksamrarn[5]

[1] Department of Physiology, Faculty of Medicine, Chiang Mai University, Chiang Mai 50200, Thailand
[2] Graduate School, Chiang Mai University, Chiang Mai 50200, Thailand
[3] Department of Chemistry, School of Science, University of Phayao, Phayao, Thailand
[4] Department of Anatomy, Faculty of Medicine, Chiang Mai University, Chiang Mai 50200, Thailand
[5] Department of Chemistry and Center of Excellence for Innovation in Chemistry, Faculty of Science, Ramkhamhaeng University, Bangkok 10240, Thailand

* **Corresponding author:** Jiraporn Tocharus, Ph D., Department of Physiology, Faculty of Medicine, Chiang Mai University, Chiang Mai 50200, Thailand, Tel.: 66 53945362, E-mail: jtocharus@gmail.com

http://dx.doi.org/10.17179/excli2019-1940

This is an Open Access article distributed under the terms of the Creative Commons Attribution License (http://creativecommons.org/licenses/by/4.0/).

EXCLI Journal 2020;19:16-32 – ISSN 1611-2156
Received: October 31, 2019, accepted: December 04, 2019, published: January 02, 2020

Supplementary Table 1: Raw data for anxiety investigation by OFT (video was tracked by SMART software; Panlab Harward Apparatus) which relate to Figure 2B–2E

OFT	Time (sec)								
Time in central	n1	n2	n3	n4	n5	n6	**Average**	**SD**	**SEM**
Control	3.13	3.14	3.81	3.05	3.87	3.11	3.36	0.39	0.16
TMF	1.51	3.49	2.6	6.21	1.56	1.56	2.53	0.99	0.40
Pioglitazone	1.91	2.03	2.31	2.66	2.23	5.89	2.08	0.21	0.08
DEX	0.06	0.41	0.2	0.2	0.57	0.03	0.22	0.18	0.07
DEX plus TMF	3.76	1.98	2.11	2.48	1.23	4.62	2.62	0.99	0.41
DEX plus pioglitazone	1.99	3.62	2.23	2.03	2.05	4.71	2.61	0.88	0.36

OFT	Time (sec)								
Time in peripheral	n1	n2	n3	n4	n5	n6	**Average**	**SD**	**SEM**
Control	296.87	295.86	296.19	296.71	295.12	296.98	296.31	0.52	0.21
TMF	293.49	298.51	297.4	297.59	293.61	291.83	296.47	2.64	1.08
Pioglitazone	295.09	297.07	296.19	293.92	298.12	296.47	296.12	0.99	0.40
DEX	299.94	298.59	299.7	299.11	298.23	299.12	299.41	0.72	0.29
DEX plus TMF	296.24	295.22	296.89	294.92	295.12	297.98	296.12	0.84	0.34
DEX plus pioglitazone	295.01	296.38	297.07	296.46	295.12	298.02	296.15	1.05	0.43

OFT	Times								
Number of rearing	n1	n2	n3	n4	n5	n6	**Average**	**SD**	**SEM**
Control	27.5	24	28	25.5	19.5	28	26.50	2.18	0.89
TMF	18	25	20	16	27.5	19.5	21.00	3.61	1.47
Pioglitazone	27	24	31	22	33	27	27.33	3.51	1.43
DEX	14	14.5	11	16	12.5	17	13.17	1.89	0.77
DEX plus TMF	24	18	20	30	19	21	20.67	3.06	1.25
DEX plus pioglitazone	30	19.5	21	22	22.5	21.5	23.50	5.68	2.32

OFT	Times								
Number of grooming	n1	n2	n3	n4	n5	n6	**Average**	**SD**	**SEM**
Control	10	8	12	14	4	8	10.00	2.00	0.82
TMF	8	7.5	11	11	5	6.5	8.83	1.89	0.77
Pioglitazone	10.5	9.5	7.5	7	8	11.5	9.17	1.53	0.62
DEX	1	2	1.5	1	2.5	1	1.50	0.50	0.20
DEX plus TMF	5	8.5	6	10	3.5	3	6.50	1.80	0.74
DEX plus pioglitazone	6.5	9.5	7	11.5	5.5	6	7.67	1.61	0.66

EXCLI Journal 2020;19:16-32 – ISSN 1611-2156
Received: October 31, 2019, accepted: December 04, 2019, published: January 02, 2020

Supplementary Table 2: Raw data for learning and memory test by MWM (video was tracked by SMART software; Panlab Harward Apparatus) which relate to Figure 3D–3F

MWM	Time (sec)								
Escape latency time	n1	n2	n3	n4	n5	n6	**Average**	**SD**	**SEM**
Control	4.89	4.69	4.14	5.18	5.94	5.53	4.57	0.39	0.16
TMF	5.32	7.2	5.79	5.24	4.38	6.01	6.10	0.98	0.40
Pioglitazone	3.76	5.41	7.01	5.79	8.79	6.32	5.39	1.63	0.66
DEX	13.38	14.65	18.17	14.43	14.56	14.03	15.40	2.48	1.01
DEX plus TMF	12.25	10.65	10.64	11.84	11.75	13.21	11.18	0.93	0.38
DEX plus pioglitazone	7.48	9.87	8.92	7.22	9.16	11.35	8.76	1.20	0.49

MWM	Time (sec)								
Time in target quadrant	n1	n2	n3	n4	n5	n6	**Average**	**SD**	**SEM**
Control	23.5	18.2	22.99	24.62	19.08	24.09	22.4	2.92	1.19
TMF	29.27	23.31	14.91	24.46	19.59	24.89	24.12	7.21	2.95
Pioglitazone	21.53	20.48	21.93	17.85	16.95	25.47	21.54	0.75	0.31
DEX	10.66	13.75	14.82	10.66	13.75	14.82	16.65	2.16	0.88
DEX plus TMF	17.95	19.42	19.52	20.57	20.96	23.26	18.21	0.88	0.36
DEX plus pioglitazone	20.49	21.01	17.49	22.1	20.73	19.41	19.31	1.90	0.78

MWM	cm/sec								
Swimming speed	n1	n2	n3	n4	n5	n6	**Average**	**SD**	**SEM**
Control	33.57	41.25	53.45	35.62	34.22	31.01	42.76	10.03	4.09
TMF	39.27	23.31	34.91	54.46	49.27	24.89	32.50	8.25	3.37
Pioglitazone	34.56	29.55	34.21	45.01	36.66	55.21	32.77	2.80	1.14
DEX	38.41	23.19	34.98	47.1	38.78	24.98	32.19	7.98	3.26
DEX plus TMF	51.13	31.17	29.52	31.59	25.31	33.26	37.27	12.03	4.91
DEX plus pioglitazone	31.12	41.61	27.81	36.91	32.31	41.78	33.51	7.20	2.94

EXCLI Journal 2020;19:16-32 – ISSN 1611-2156
Received: October 31, 2019, accepted: December 04, 2019, published: January 02, 2020

Supplementary Table 3: Raw data to investigate amyloidogenesis genes expression by qRT-PCR which relate to Figure 4A–4D

qRT-PCR	Fold change								
ADAM10	n1	n2	n3	n4	n5	n6	**Av-er-age**	**SD**	**SEM**
Control	1	1	1	1	1	1	1.00	0.00	0.00
TMF	0.833409	1.062206	1.184229	0.833409	1.062206	1.184229	1.03	0.18	0.07
Pioglitazone	1.773053	0.8704219	0.8440408	1.773053	0.8704219	0.8440408	1.16	0.53	0.22
DEX	0.2477358	0.1157734	0.2160244	0.4477358	0.3157734	0.4160244	0.19	0.07	0.03
DEX plus TMF	0.8410285	0.7429374	1.264839	0.7410285	0.8429374	1.464839	0.95	0.28	0.11
DEX plus pioglitazone	1.911946	1.329108	1.694478	1.911946	1.329108	1.694478	1.65	0.29	0.12

qRT-PCR	Fold change								
BACE1	n1	n2	n3	n4	n5	n6	**Av-er-age**	**SD**	**SEM**
Control	1	1	1	1	1	1	1.00	0.00	0.00
TMF	1.125341	0.2770034	1.197147	1.125341	0.2770034	1.197147	0.87	0.51	0.21
Pioglitazone	0.6465945	0.1194291	0.6414075	0.6465945	0.1194291	0.6414075	0.47	0.30	0.12
DEX	2.197812	1.481598	1.563029	2.197812	1.481598	1.563029	1.75	0.39	0.16
DEX plus TMF	1.595159	0.8679951	1.252444	1.295159	0.8679951	1.252444	1.24	0.36	0.15
DEX plus pioglitazone	0.7254966	0.8641342	0.9435608	0.7254966	0.8641342	0.9435608	0.84	0.11	0.05

qRT-PCR	Fold change								
PS1	n1	n2	n3	n4	n5	n6	**Aver-age**	**SD**	**SEM**
Control	1	1	1	1	1	1	1.00	0.00	0.00
TMF	0.5703976	1.758815	0.5686346	0.5703976	1.758815	0.5686346	0.97	0.69	0.28
Pioglita-zone	0.6068053	0.3626619	0.5214976	0.6068053	0.3626619	0.5214976	0.50	0.12	0.05
DEX	1.57512	1.570488	2.214808	1.57512	1.570488	2.214808	1.79	0.37	0.15
DEX plus TMF	1.663366	1.655074	0.8003142	1.663366	1.655074	0.8003142	1.37	0.50	0.20
DEX plus pioglita-zone	1.179073	0.8606647	0.6428713	1.179073	0.8606647	0.6428713	0.89	0.27	0.11

qRT-PCR	Fold change								
APP	n1	n2	n3	n4	n5	n6	**Av-er-age**	**SD**	**SEM**
Control	1	1	1	1	1	1	1.00	0.00	0.00
TMF	1.145007	1.077904	0.9897956	1.145007	1.077904	0.9897956	1.07	0.08	0.03
Pioglita-zone	0.9769166	1.095183	0.9521693	0.9769166	1.095183	0.9521693	1.01	0.08	0.03
DEX	1.145753	1.44271	1.280274	1.145753	1.44271	1.280274	1.29	0.15	0.06
DEX plus TMF	1.175246	1.950779	1.086286	1.175246	0.9507794	1.086286	1.40	0.48	0.19
DEX plus pioglitazone	1.276485	1.276485	0.9233383	1.276485	1.276485	0.9233383	1.16	0.20	0.08

EXCLI Journal 2020;19:16-32 – ISSN 1611-2156
Received: October 31, 2019, accepted: December 04, 2019, published: January 02, 2020

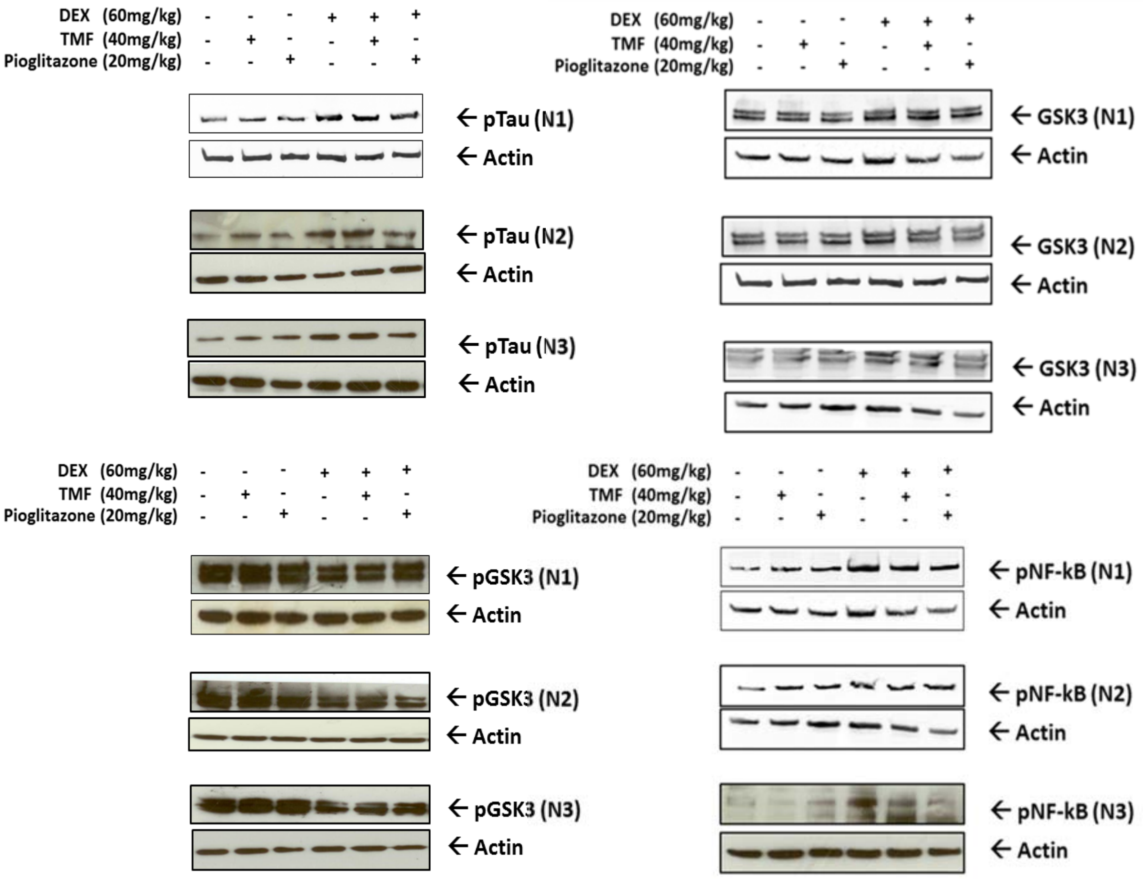

Supplementary Figure 1: Triplicate data for Western blotting experiments to study Tau protein expression which relate to Figure 5B–5E

Supplementary Table 4: Raw data for neuronal cell apoptosis study by TUNEL assay which relate to Figure 6C

TUNEL	Apoptosis (% of control)						
	n1	n2	n3	n4	**Average**	**SD**	**SEM**
Control	100	100	100	100	100.00	0.00	0.00
TMF	94.11765	94.11765	70.58823	47.05882	86.27	13.58	6.79
Pioglitazone	94.11765	117.6471	141.1765	70.58823	117.65	23.53	11.76
DEX	188.2353	235.2941	208.2353	211.7647	210.59	23.62	11.81
DEX plus TMF	164.7059	141.1765	211.7647	141.1765	172.55	35.94	17.97
DEX plus pioglitazone	70.58823	117.6471	188.2353	164.7059	125.49	59.21	29.61

EXCLI Journal 2020;19:16-32 – ISSN 1611-2156
Received: October 31, 2019, accepted: December 04, 2019, published: January 02, 2020

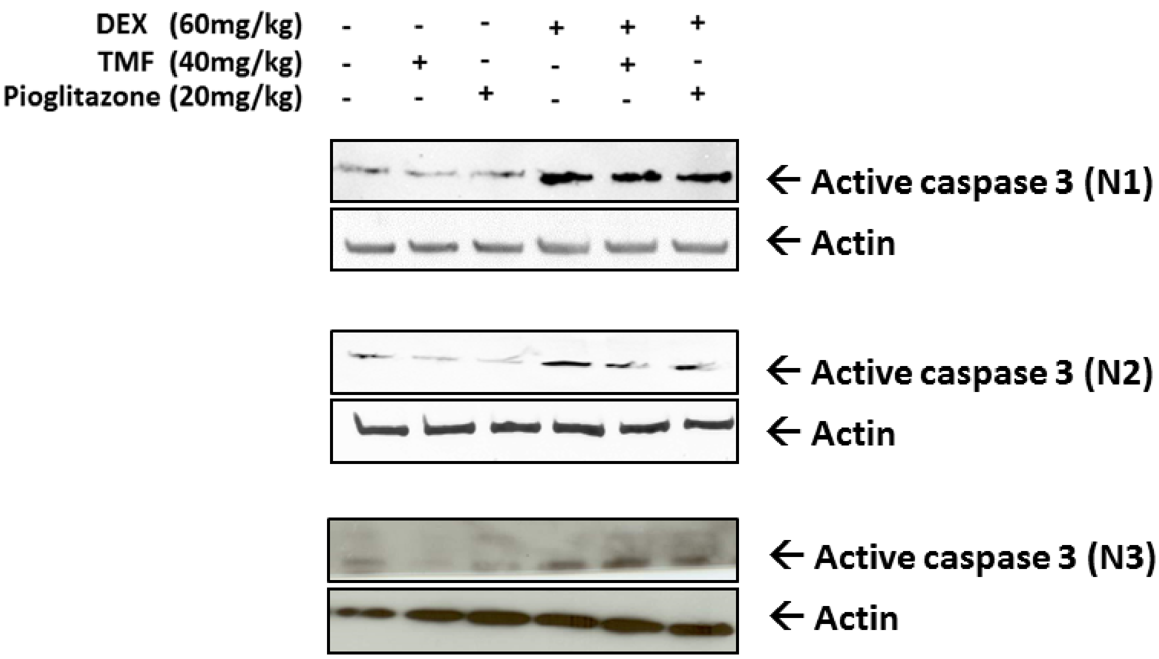

Supplementary Figure 2: Triplicate data for Western blotting experiments to study neuronal apoptosis which relate to Figure 6D.

Supplementary Table 5: Raw data to investigate AChE activity which relate to Figure 7A

AchE activity	mmol ACTC min-1mg protein-1								
	n1	n2	n3	n4	n5	n6	**Av-er-age**	**SD**	**SEM**
Control	0.089964	0.088494	0.032928	0.045697	0.048363	0.079821	0.07	0.03	0.01
TMF	0.028371	0.037044	0.127743	0.088494	0.027048	0.04263	0.06	0.06	0.02
Pioglitazone	0.034545	0.045423	0.003381	0.100254	0.070854	0.034251	0.03	0.02	0.01
DEX	0.155085	0.114072	0.125852	0.136837	0.146265	0.116865	0.13	0.02	0.01
DEX plus TMF	0.103822	0.081438	0.053891	0.098891	0.072757	0.081879	0.08	0.03	0.01
DEX plus pioglitazone	0.014406	0.08613	0.074525	0.065288	0.085995	0.053059	0.06	0.04	0.01

EXCLI Journal 2020;19:16-32 – ISSN 1611-2156
Received: October 31, 2019, accepted: December 04, 2019, published: January 02, 2020

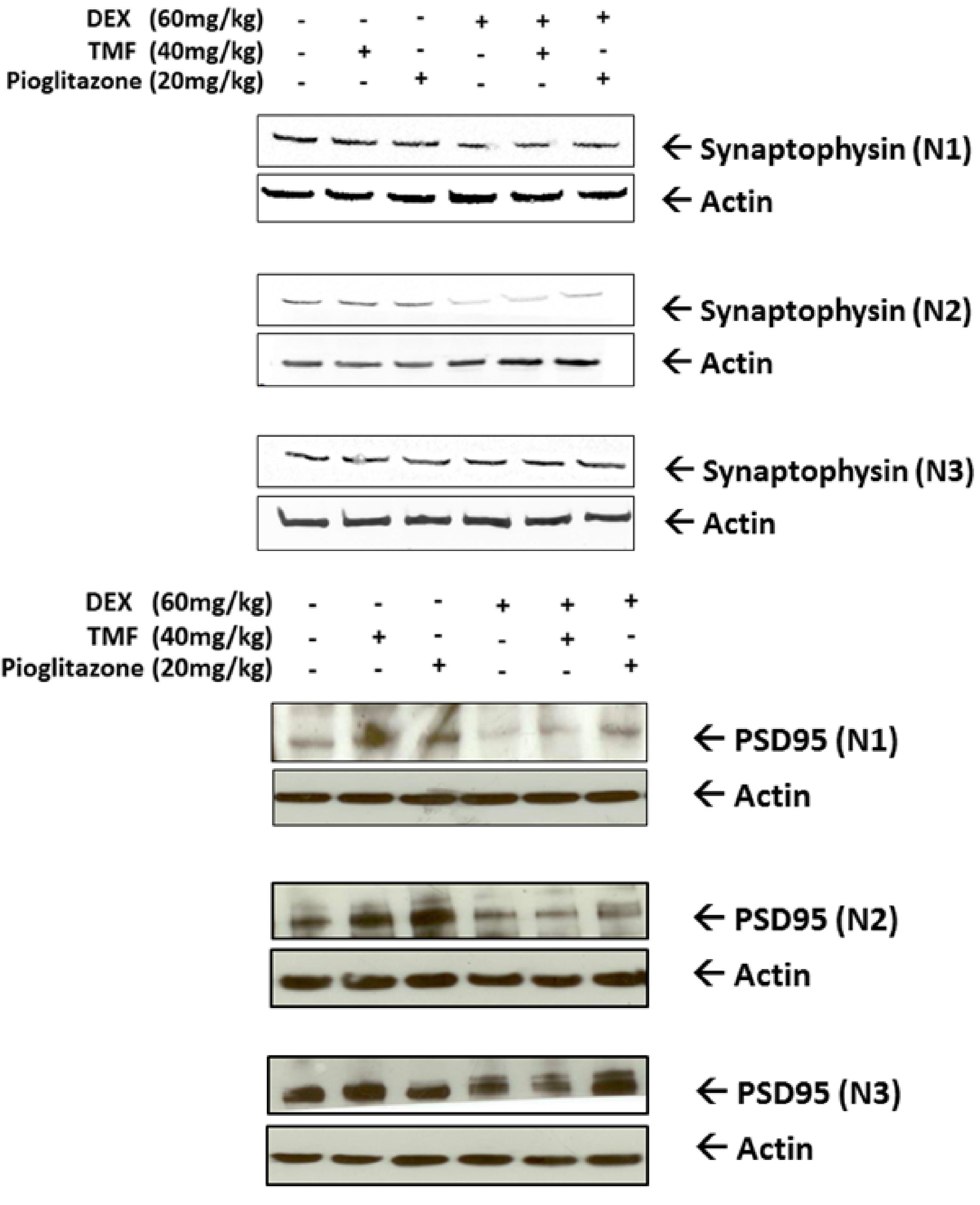

Supplementary Figure 3: Triplicate data for Western blotting experiments to study neuronal synaptic function which relate to Figure 6D
